# A Comprehensive Overview of the Neural Mechanisms of Light Therapy

**DOI:** 10.1007/s12264-023-01089-8

**Published:** 2023-08-09

**Authors:** Xiaodan Huang, Qian Tao, Chaoran Ren

**Affiliations:** 1 Guangdong-Hongkong-Macau Institute of CNS Regeneration, Ministry of Education CNS Regeneration Collaborative Joint Laboratory, Jinan University, Guangzhou, 510632 China; 2 Psychology Department, School of Medicine, Jinan University, Guangzhou, 510632 China

**Keywords:** Light therapy, Neural circuit, Mood, Cognition, Pain, Sleep, Development, Metabolism

## Abstract

Light is a powerful environmental factor influencing diverse brain functions. Clinical evidence supports the beneficial effect of light therapy on several diseases, including depression, cognitive dysfunction, chronic pain, and sleep disorders. However, the precise mechanisms underlying the effects of light therapy are still not well understood. In this review, we critically evaluate current clinical evidence showing the beneficial effects of light therapy on diseases. In addition, we introduce the research progress regarding the neural circuit mechanisms underlying the modulatory effects of light on brain functions, including mood, memory, pain perception, sleep, circadian rhythm, brain development, and metabolism.

## Introduction

Changes in lighting conditions have broad effects on diverse physiological and behavioral functions, including circadian rhythm, mood, and cognition [1, 2]. In humans, light therapy can alleviate depression, promote cognitive function, and relieve pain symptoms [3–12]. It provides a solution for the treatment of brain diseases with the advantages of non-invasiveness, few side-effects, and low cost. Given the acknowledgment of its beneficial effects on brain diseases, the neural mechanisms underlying the beneficial effects of light therapy on brain functions are not well understood. This has caused obstacles to the application and optimization of light therapy in the clinic. Here, we review the progress of human clinical research into light therapy and introduce the basic research progress regarding the circuit mechanisms underlying the modulatory effects of light on brain functions. Through analyzing these research findings, we hope to provide readers with a comprehensive understanding of the modulatory effects of light on brain functions and inspire new ideas for the application and optimization of light therapy.

## Clinical Evidence for Light Therapy

### Light Therapy for Depression

As early as the 1980s, light therapy was first used to treat seasonal affective disorder (SAD) in clinical settings. The results of a meta-analysis that included 19 randomized controlled trials confirmed the effectiveness of light therapy in the treatment of SAD (*d* = −0.37, 95% CI: −0.63, −0.12) [13]. As a cost-effective physical intervention, light therapy has many advantages, such as high safety, mild side-effects, and convenience. To date, light therapy has become the first-line treatment for SAD. In addition to SAD, an increasing number of clinical studies have indicated that light therapy has certain therapeutic effects on various types of non-seasonal depression. For instance, Lam and colleagues conducted a randomized controlled trial on 122 patients with major depressive disorder, and the results showed that the light therapy group (*d* = 0.80, 95% CI: 0.28, 1.31) and the combined treatment group (light therapy/fluoxetine) (*d* = 1.11, 95% CI: 0.54, 1.64) had significantly superior improvements in outcomes compared to those in the placebo group [14]. Pierre and colleagues conducted a systematic review of major depressive disorder and concluded that light therapy and antidepressants were equally effective (SMD = 0.19, 95% CI: −0.08, 0.45) and that light therapy combined with antidepressants was significantly better than monotherapy with antidepressants (SMD = 0.56, 95% CI: 0.24, 0.88) [15]. An open trial suggested that 3 weeks of bright light therapy is efficacious for antepartum depression, and the benefits were seen at the 2-week follow-up assessment [16]. Our previous study also suggested that 8 weeks of bright light therapy had beneficial effects in treating subthreshold depression in a college student sample [17]. In summary, the beneficial effects of light therapy have been confirmed in several types of depression, but the optimal parameters for this intervention are controversial. However, guidelines for the clinical applications of light therapy in depression are lacking.

### Light Therapy for Cognition

Light is essential for many cognitive tasks. A Cochrane meta-study that included three randomized controlled trials and two before-after trials with 282 daytime worker participants, showed that bright illumination in the environment improves alertness [18]. Our recent meta-study showed that light exposure in laboratory settings improves both objective and subjective alertness, and the correlated color temperature of light treatment is a key parameter to determine the beneficial effects [19]. The potential application of light therapy for cognitive disorders has been increasingly recognized in the last decade [20]. A recent randomized placebo-controlled trial of blue wavelength exposure was conducted in 32 adults with mild traumatic brain injury [21]. Compared with the placebo, blue light led to reduced daytime sleepiness and improved executive functioning, which was associated with brain changes in the retinohypothalamic system [21]. A Cochrane meta-study found no adequate evidence of the effectiveness of light therapy in managing cognitive function in dementia [22]. A recent study using a crossover design suggested that a whole-day lighting scheme that follows the natural light/dark cycle improves cognitive performance in older adults, as indexed by the trail-making test (assessing executive function) and the digit symbol substitution test (assessing processing speed and attention) [23]. Another recent clinical trial demonstrated the efficacy of bright light therapy in improving objective cognitive function in the survivors of hematopoietic stem cell transplants [24]. Blue-enriched light therapy has been found to be beneficial in improving general cognitive function in patients with mild and moderate Alzheimer’s disease (AD) [25]. Overall, it seems that light intervention is useful for improving alertness in healthy individuals, but the evidence for its effect on other cognitive functions, such as memory, attention, and executive function, is not adequate. Investigations on the effectiveness of light therapy in patients with cognitive disorders are scarce, and conflicting results have been reported. We speculate that at least three reasons contribute to these inconsistent results. First, the stage and severity of cognitive impairment should be considered. Second, outcomes should be expanded to many cognitive domains, rather than general cognition. Finally, light therapy may be a good supplementary intervention to traditional cognitive interventions. In the future, more studies are required to investigate the efficacy of light therapy on different aspects of cognition.

### Light Therapy for Pain

In clinical practice, the common approaches to treating chronic pain include opioid analgesic medication [26], cognitive behavioral therapy [27, 28], meditation, and acupuncture [29, 30]. However, these approaches have various limitations. For instance, pain treatments are not always effective, have side-effects, and are expensive [31]. Although clinical evidence documenting the relationship between light therapy and pain is scarce, light therapy is a promising, available, and safe intervention to manage chronic pain. A prospective study evaluated the effect of natural sunlight on pain among patients undergoing spinal surgery [9]. The results showed that patients staying on the bright side of the room had decreased pain, analgesic medication use, and pain medication cost than those staying on the dim side [9]. Patients with fibromyalgia and US military veterans often report experiencing pain; Burgess and colleagues conducted three studies on the effect of bright light therapy in these two populations. Their first study indicated that bright light therapy improves pain sensitivity (less sensitive to pain) in female patients with fibromyalgia [10]. The second study suggested that morning bright light therapy reduces pain intensity, pain behavior, and the thermal pain threshold in US veterans with chronic low back pain [11]. Importantly, phase advances in circadian timing were significantly associated with increased pain tolerance in both studies. A further study evaluated volatility and showed that bright light therapy was related to participants experiencing fewer “pain flares” [32]. Overall, several limitations, such as small sample sizes and the lack of a double-blind design, were noted for the above studies. More future clinical studies of high quality are needed to document the evidence for light therapy and pain.

### Light Therapy for Sleep and Circadian Rhythms

Light, sleep, and circadian rhythms have closely interacted to help organisms to adapt to the environment [2]. First, light influences the suprachiasmatic nucleus (SCN) that controls circadian rhythms. Second, light suppresses melatonin secretion, which is crucial for the regulation of circadian rhythms and sleep. Consequently, light therapy has been applied as a potential treatment for sleep disorders. For instance, a randomized control study suggested that light and dark exposure in shift work nurses improves their insomnia symptoms [33]. Morning bright light exposure advances the circadian rhythms in patients with insomnia [34] and delays sleep phase syndrome [35]. However, one study reported limited effects of light therapy on individuals >55 years old with mild early-morning awakenings [36]. A meta-study that included 53 studies and 1154 participants found beneficial effects of light therapy on sleep problems in general, including insomnia, and the sleep problems associated with AD and dementia, but limited effects on circadian rhythm sleep disorders [37]. Furthermore, combined bright light and melatonin therapies have been shown to be superior to single therapy in advancing the phase in healthy individuals and improving sleep outcomes in elderly populations with cognitive decline [38]. Light therapy also improves sleep quality in patients with Parkinson’s disease [39]. It has been noted that the parameters of light therapy greatly influence the sleep outcomes and circadian rhythms. A consistent finding is that light exposure during daytime has beneficial effects on sleep, whereas light exposure at night has adverse effects. More investigations are needed to explore the effects of light characteristics on sleep and circadian rhythms.

## Human Neuroimaging Studies Related to Light Therapy

**Fig. 1:**
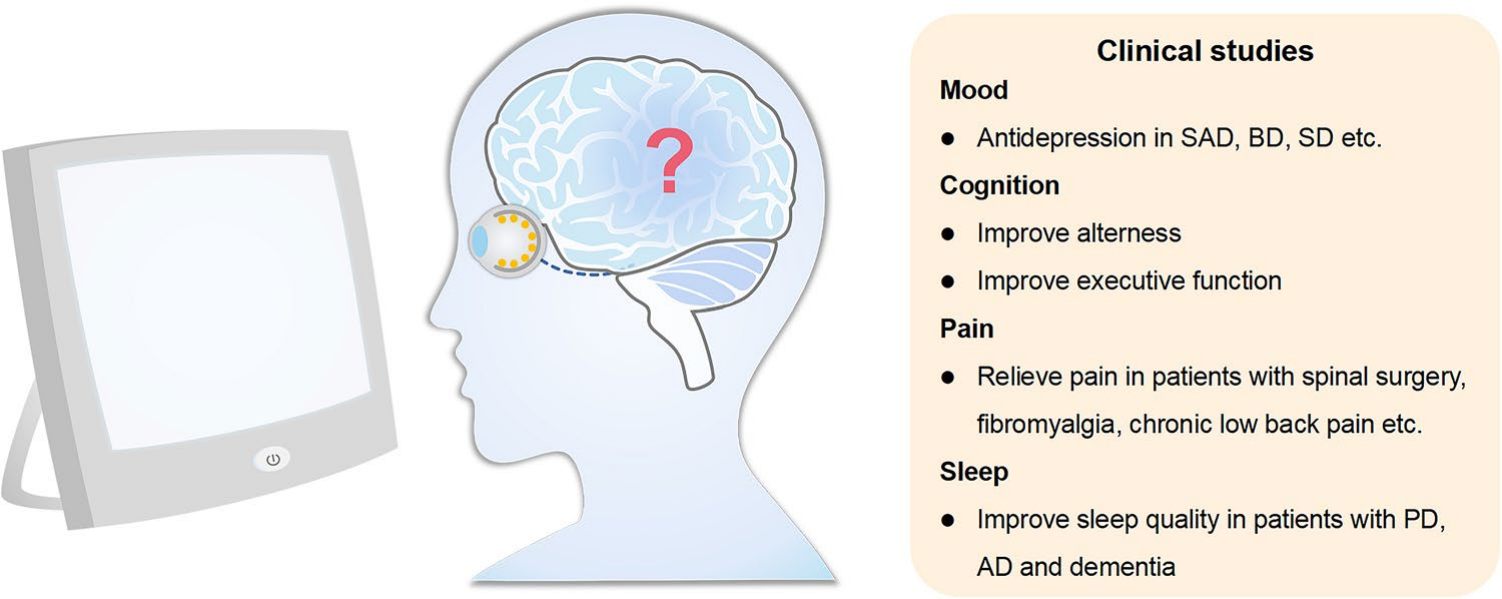
Clinical studies of light therapy. The clinical studies of light therapy in the regulation of mood, cognition, and pain. SAD, seasonal affective disorder; BD, bipolar disorder; SD, subthreshold depression; PD, Parkinson's disease; AD, Alzheimer’s disease.

Although light therapy has been widely reported to be beneficial in clinical studies (Fig. 1), the underlying mechanisms remain largely unknown. The clinical practice of light therapy in the treatment of depression is mainly based on the phase-shifting hypothesis, which indicates that light has a role in re-synchronizing circadian rhythms [40]. However, other mechanisms may also mediate the antidepressant effect of light therapy based on three lines of evidence. First, light therapy requires a higher intensity (>5000 lx) to treat depression than to alter circadian rhythms (~120 lx). Second, the relief of depressive symptoms is not consistently associated with changes in circadian rhythm [40, 41]. Finally, light therapy has been shown to be efficacious in treating not only seasonal depression but also many types of non-seasonal depression [14, 42–45]. Pertinently, not all types of non-seasonal depression are related to phase shifting.

Neuroimaging techniques have provided some clues for the neural mechanisms underlying the beneficial effects of light therapy. An early study collected functional magnetic resonance imaging (fMRI) data while participants attended to auditory and visual stimuli in darkness following short-term light exposures in the scanner [46]. The results revealed increased activation in the occipito-parietal network and decreased activation in the hypothalamus [46]. By using an auditory oddball task, another fMRI study found that short-term exposure to bright white light induced dynamic responses in the posterior thalamus and subcortical regions supporting attentional effects [47]. A further fMRI study evaluated the wavelength effect of light exposure on an auditory working memory task [48]. Compared with green light exposure, blue light exposure led to changes in the left intraparietal sulcus, supramarginal gyrus, right insula, left middle frontal gyrus, and left thalamus [48]. All of the above studies demonstrated non-visual responses to short-term light exposure in the human brain within scanners. However, neuroimaging studies on light therapy are few. We only identified two relevant studies. Fisher and colleagues conducted an fMRI study on healthy individuals with an emotional faces paradigm [49]. The results demonstrated that a three-week bright-light intervention changed amygdala-prefrontal reactivity and functional coupling, which was partly moderated by the 5-HTTLPR (serotonin-transporter-linked promoter region) genotype [49]. This important finding has provided the first evidence indicating that serotonin and the threat-related circuit may underlie the effects of light therapy in humans. Another study used resting-state fMRI to investigate the neural mechanisms underlying two-week morning bright light exposure in individuals with sleep disturbances [50]. The results demonstrated decreased functional connectivity in the anterior insular and frontal opercular regions that were correlated with decreased sleep latency in a bright light group [50]. More longitudinal neuroimaging studies are needed to reveal the neural mechanisms underlying the non-visual effects of light.

## Animal Studies

As noted above, clinical studies of light therapy have mainly focused on the utility of this treatment for mood-, cognition-, pain-, and sleep-related disorders. Due to the complexity of the biological mechanisms underlying these disorders and the limitations of technologies that can be used in humans, the neural mechanisms of light therapy are poorly understood. To further analyze the neural mechanism of light therapy, experimental animals are indispensable research subjects. Unveiling the neuronal basis for the effects of light on brain functions in animal models constitutes a promising step toward new treatments for neuropsychiatric disorders.

### Photosensitive Cells in the Retina

In mammals, the effects of external light on brain functions are mainly mediated by specific visual circuits [51]. At the beginning of the visual circuits, photosensitive cells in the retina convert light information into bioelectrical information that is transmitted to different brain regions [51–53]. In the mammalian retina, there are three types of light-sensitive cells: rods, cones, and intrinsic photosensitive retinal ganglion cells (ipRGCs) [54, 55]. Among these, rods and cones are conventional photoreceptors that transmit light signals to RGCs through bipolar cells. Owing to the expression of the photosensitive protein melanopsin, ipRGCs not only receive light signals transmitted from conventional photoreceptors but also respond directly to external light stimuli even in the absence of rods and cones [55–60]. Studies have found that ipRGCs are highly conserved in several species, including humans [52, 61, 62], and they transmit light information to multiple brain regions to regulate brain functions unrelated to image-forming vision, including the circadian rhythm [1], cognition [20], and mood [2]. The discovery of ipRGCs also opened up a new field of research, namely, the non-image-forming visual functions of light.

**Fig. 2:**
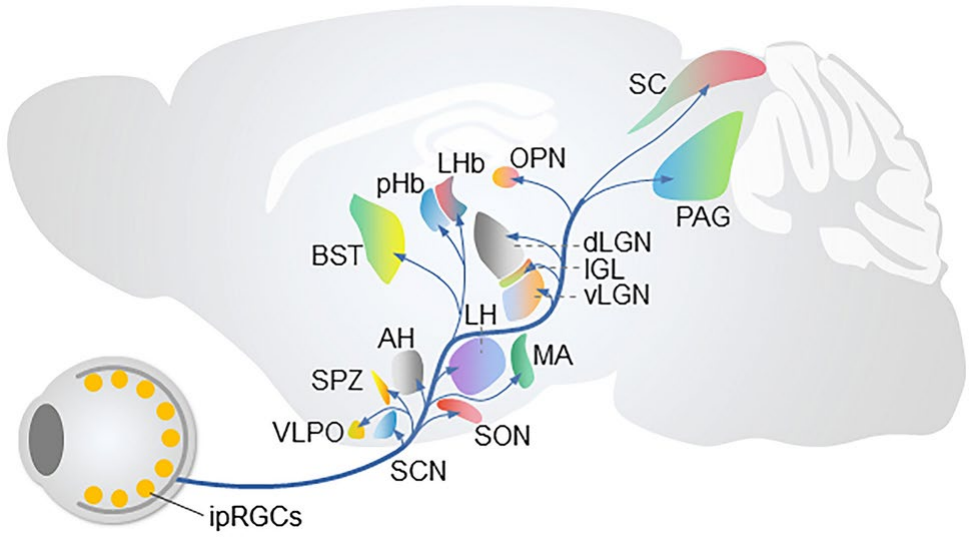
The efferent projections of ipRGCs. The main brain targets of ipRGCs. IPL, inner plexiform layer; ipRGCs, intrinsic photosensitive retinal ganglion cells; AH, anterior hypothalamus; BST, bed nucleus of the stria terminalis; SPZ, subparaventricular zone; VLPO, ventrolateral preoptic area; SON, supraoptic nucleus; SCN, suprachiasmatic nucleus; MA, medial amygdaloid nucleus; pHb, perihabenular nucleus; LHb, lateral habenula; SC, superior colliculus; PAG, periaqueductal gray; OPN, olivary pretectal nucleus; dLGN, dorsal lateral geniculate nucleus; IGL, intergeniculate leaflet; vLGN, ventral lateral geniculate nucleus.

IpRGCs can be divided into different subtypes according to their morphological characteristics, such as the size of the somata, the ramification pattern of dendrites stratified in the inner plexiform layer of the retina, and the size and complexity of the dendritic field. At present, it is known that mouse ipRGCs are divided into six subtypes, M1-M6 [52]; rat ipRGCs are divided into five subtypes, M1-M5 [61], and human ipRGCs are known to have four subtypes, M1-M4 [62]. In addition to morphological differences, ipRGCs vary in other aspects. For example, M1-ipRGCs have the highest level of melanopsin expression, while M4-ipRGCs express melanopsin at a very low level [63, 64]. In addition, different subtypes of ipRGC differ in their response to light and their projection patterns to brain regions [61, 65, 66] (Fig. 2). It is precisely because of the cellular heterogeneity of ipRGCs that they mediate the effects of light on diverse brain functions, such as mood, memory, pain perception, sleep, circadian rhythm, brain development, and metabolism.

### Circuit Mechanisms Underlying the Effects of Light on Mood

Clinical studies of SAD patients have found that rapid tryptophan depletion reverses the antidepressant effects of light therapy [67–69], suggesting that light information might influence depressive-like behaviors through specific circuits linking the retina and the midbrain monoaminergic centers. The lateral habenula (LHb), part of the epithalamus, is a highly conserved nucleus across species, and it regulates the flow of information from the limbic system to the midbrain monoaminergic centers [70]. Studies of rodents have suggested that light influences neural activity in the LHb [2, 71]. Therefore, visual circuits related to the LHb might mediate the effects of light on depressive-like behaviors. Consistent with this view, our recent study of mice found that the ventral lateral geniculate nucleus and intergeniculate leaflet (vLGN/IGL) are important relay regions linking the retina and LHb [72]. Bright light signals transmitted by a subset of M4-ipRGCs activate a subset of GABAergic neurons in the vLGN/IGL, which in turn inhibit the activity of excitatory neurons in the LHb. We found that specific activation of RGCs projecting to the vLGN/IGL, activation of LHb-projecting vLGN/IGL neurons, or inhibition of postsynaptic LHb neurons is sufficient to decrease the depressive-like behaviors evoked by long-term exposure to aversive stimuli or chronic social defeat stress. Furthermore, we demonstrated that the activation of the retina-vLGN/IGL-LHb pathway is needed for the anti-depressive effects of bright light treatment [72]. These results provide a potential mechanistic explanation for the antidepressant effects of bright light treatment.

Unlike the role of the LHb in mediating the beneficial effects of light on depressive-like behaviors, recent studies have shown that the perihabenular nucleus (pHb) located in the dorsal thalamus mediates the negative effects of light on depressive-like behaviors [73, 74]. For example, Fernandez and colleagues found that long-term exposure to an ultradian light : dark cycle (T7 cycle : 3.5 h light and 3.5 h dark) accompanied by pHb activation increases depressive-like behaviors in mice [73]. They further demonstrated that the depression-inducing effects of a fast ultradian light cycle are mediated by an M1-ipRGC→pHb→mPFC pathway. In addition to the ultradian light/dark cycle, An and colleagues found that excessive light exposure at night (LAN) also increases depressive-like behaviors without disrupting the circadian rhythm [74]. Importantly, An and colleagues demonstrated that the depression-inducing effects of LAN are mediated by a visual circuit consisting of M1-ipRGCs, the pHb, and the nucleus accumbens. Those two studies provide direct evidence that the pHb-related visual circuits play a pivotal role in mediating the negative effects of light on depressive-like behaviors.

**Fig. 3:**
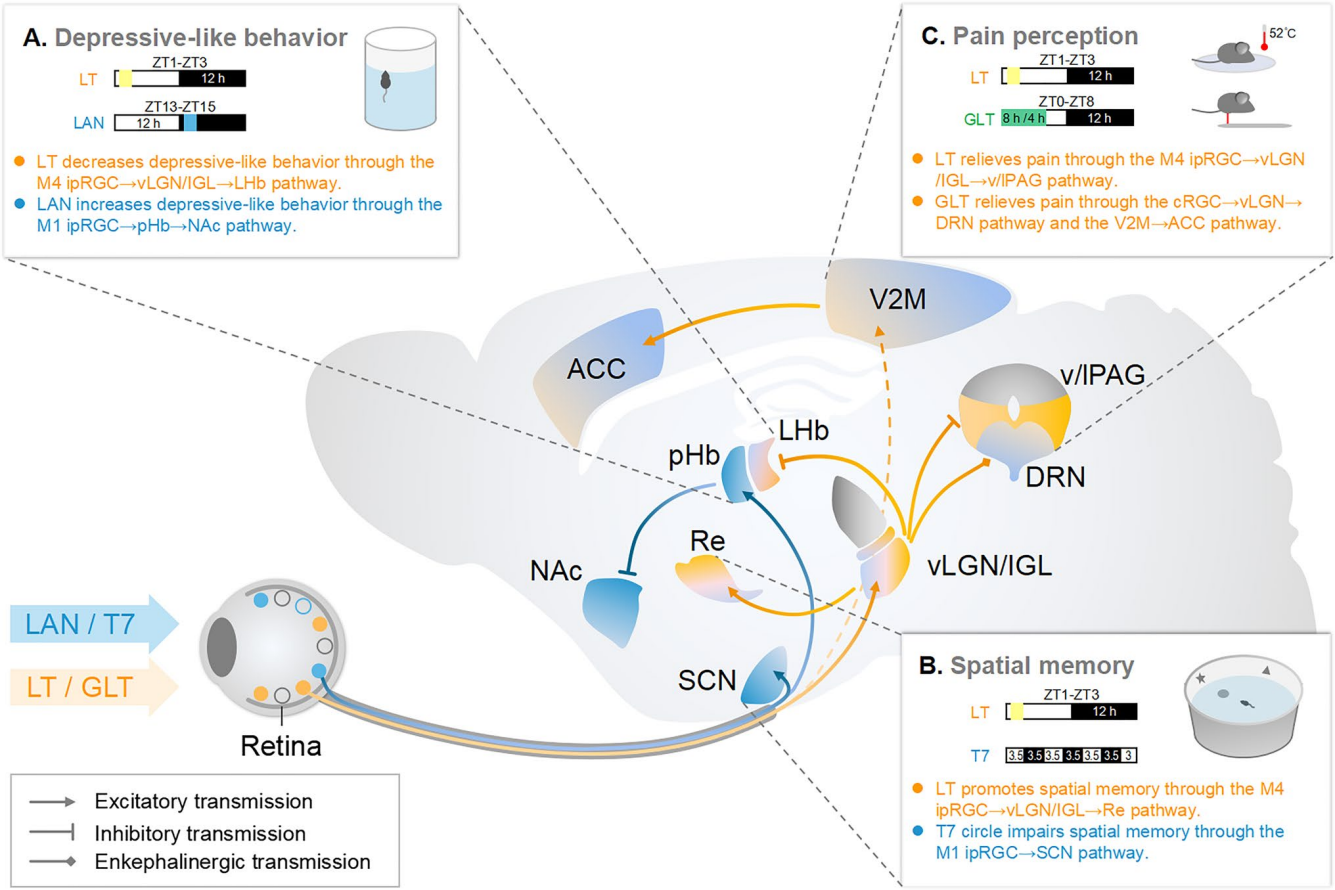
Circuit mechanisms underlying the effects of light treatment. **A** Bright light treatment (LT) in the daytime induces antidepressant effects through the M4 ipRGC→vLGN/IGL→LHb pathway (orange), while light at night (LAN) increases depressive-like behaviors through the M1 ipRGC→pHb→NAc pathway (blue). **B** LT promotes spatial memory through the M4 ipRGC→vLGN/IGL→Re pathway (orange), while a fast ultradian light cycle (T7, 3.5 h light, and 3.5 h dark) impairs memory (blue) through the M1 ipRGC→SCN pathway. (C) LT exerts antinociceptive effects through the M4 ipRGC→vLGN/IGL→v/lPAG pathway, while green light treatment (GLT) has antinociceptive effects through the cRGC→vLGN→DRN pathway and V2M→ACC pathway. RGC, retinal ganglion cell; vLGN/IGL, ventral lateral geniculate nucleus and intergeniculate leaflet; PHb, perihabenular nucleus; LHb, lateral habenula; NAc, nucleus accumbens; SCN, suprachiasmatic nucleus; Re, reunions nucleus; v/lPAG, lateral and ventral lateral periaqueductal gray; DRN, dorsal raphe; V2M, the secondary visual cortex; ACC, anterior cingulate cortex.

However, the relevant question is about the idea that plenty of light during the daytime should also activate the M1-ipRGC→pHb pathway, which can increase depressive-like behaviors. Why does daytime light exposure (such as bright light therapy) have an antidepressant effect? One possible explanation is that the excitability of pHb neurons is higher at night than in the daytime and tends to conduct nighttime light information [74]. Taken together, recent studies conducted in rodents have suggested that the pHb acts as a valve controlled by the circadian rhythm, specifically mediating the regulation of negative mood by nocturnal light messages, and daytime light exposure alleviates depression through the LHb-related visual circuits (Fig. 3A).

### Circuit Mechanisms Underlying the Effect of Light on Memory

Memory dysfunction is a typical characteristic of AD, the pathogenesis of which is complex, including amyloid-β (Aβ) deposition, tau accumulation, and disrupted network oscillation [75, 76]. Studies conducted in mice have shown that light flickering at 40 Hz (gamma entrainment using sensory, GENUS) drives gamma oscillations in the visual cortex [77]. The authors demonstrated that the long-term application of GENUS improves memory in several mouse models of AD, including the CK-p25, P301S, and 5xFAD models [78]. In addition, by training neurons to oscillate at gamma frequency, GENUS triggers the engulfment activity of microglia, while decreasing Aβ deposition and tau accumulation in several brain regions, including the visual cortex, hippocampus (HPC), and prefrontal cortex (PFC) [77, 78]. Moreover, GENUS enhances synaptic function in neurons [78]. These findings uncover a previously unappreciated function of gamma rhythms in recruiting both neural and glial responses to attenuate AD-associated pathology. However, the circuit mechanisms underlying the effects of GENUS on memory are still unknown. It should also be noted that a recent study found that 40 Hz flickering light does not entrain gamma oscillations and suppress Aβ in the brains of APP/PS1 and 5xFAD mouse models of AD [79]. Thus, more detailed work is needed to investigate the effects of GENUS and the underlying mechanism.

Accumulating evidence suggests that bright light has beneficial effects on memory. In humans, brighter illumination during the day improves cognitive performance [4–8], and bright light therapy appears to attenuate cognitive deterioration in early-stage dementia [22, 80]. In rodents, bright light has been shown to enhance fear and spatial memory [81–83]. However, the neural mechanisms underlying the effects of bright light on memory are not well understood. A recent study by our group probed the circuit mechanisms underlying the beneficial effects of bright light treatment on spatial memory [84]. We found that long-term exposure to bright light promotes the spatial memory tested in the novel location test and Morris water maze test in young WT mice (3 months old); this is accompanied by the increased power of gamma oscillation in the HPC during the performance of a spatial memory-related task [84]. These results suggest that bright light treatment might promote spatial memory through certain visual circuits associated with the HPC. However, it is well established that the HPC does not receive direct retinal projections. To unveil the neural circuits underlying the beneficial effects of bright light on spatial memory, we conducted whole-brain c-Fos mapping to determine the neural substrates that can be regulated by bright light. We found that bright light increases c-Fos expression in the nucleus reunions (Re) [84], which interacts with the HPC and plays an important role in the regulation of memory [85–87]. These results suggest that the spatial-memory-promoting effects of bright light might be mediated by a visual circuit linking the retina and Re. Consistent with this view, we found that bright light promotes spatial memory through activating an M4-ipRGC→vLGN/IGL→Re pathway [84] (Fig. 3B). These results reveal a dedicated subcortical visual circuit that mediates the spatial-memory-promoting effects of bright light treatment.

It should be noted that both GENUS and bright light promote spatial memory and increase gamma oscillation in the HPC. One might expect that bright light should also improve spatial memory in a mouse model of AD. However, we found that long-term exposure to bright light does not significantly promote spatial memory in 6-month-old 5xFAD mice [84]. In addition, unlike GENUS, bright light alone does not directly increase gamma oscillation in the HPC [84]. The HPC gamma oscillation detected during the spatial memory-related task may be too weak to reduce the amyloid load and reverse the deficits in spatial memory of 5xFAD mice. On the other hand, GENUS does not show beneficial effects on spatial memory in WT mice [78], suggesting that the neural mechanisms underlying the spatial-memory-promoting effects of bright light treatment and GENUS are different.

In addition to the beneficial effects of light on memory, certain light patterns can impair memory. Fernandez and colleagues found that a fast ultradian light cycle (T7) not only increases depressive-like behaviors but also results in memory deficits, accompanied by decreased cellular plasticity in the HPC [73]. They demonstrated that the negative effects of the fast ultradian light cycle on memory are mediated by an M1-ipRGC→SCN pathway (Fig. 3B). Given that the SCN plays a pivotal role in the regulation of circadian rhythm, these results suggest that a disrupted circadian rhythm might underlie fast ultradian light cycle-induced memory deficits. In addition, studies conducted in grass rats, a diurnal rodent species, found that dim light exposure (5 lx) at night impairs spatial memory accompanied by a decreased dendritic length in the dentate gyrus and basilar CA1 dendrites [88], without affecting the circadian rhythm tested in the wheel-running test. This result suggests that certain visual circuits unrelated to the regulation of circadian rhythm might also mediate the negative effects of LAN on memory.

### Circuit Mechanisms Underlying the Effect of Light on Pain Perception

Clinical studies have shown that light therapy can relieve pain symptoms in patients with chronic low back pain, headache, fibromyalgia, and postoperative pain [9–12]. Consistent with this, animal studies have shown that light also has antinociceptive effects in rodents [89, 90]. However, the precise circuits that mediate the effects of light on nocifensive behaviors remain unclear. Our recent study in mice found that GABAergic neurons in the vLGN/IGL directly synapse with GABAergic neurons in the lateral and ventral lateral periaqueductal gray (l/vlPAG) [91], which is an important part of the ascending pain conduction and descending pain regulation systems [92–95]. We found that specific activation of the vLGN/IGL not only induces inhibitory postsynaptic currents in l/vlPAG GABAergic neurons but also reduces the excitatory effects of pain-related stimuli on l/vlPAG GABAergic neurons [91], suggesting that the vLGN/IGL→l/vlPAG pathway might modulate pain-related behaviors. Consistent with this, we found that specific activation of l/vlPAG-projecting vLGN/IGL neurons or inhibition of l/vlPAG postsynaptic neurons not only elevates the pain threshold in WT mice but also improves the pain-related symptoms in mouse models of pain [91], indicating the antinociceptive effects induced by activation of the vLGN/IGL→l/vlPAG pathway. We further found that the vLGN/IGL→l/vlPAG pathway receives direct innervation from RGCs [91], suggesting that light information transmitted by the retina→vLGN/IGL→l/vlPAG pathway might modulate pain-related behaviors. In support of this view, we demonstrated that the antinociceptive effects of bright light treatment are dependent on the activation of the retina→vLGN/IGL→l/vlPAG pathway [91] (Fig. 3C).

In addition to bright light, exposure to green light also has antinociceptive effects in both humans and rodents [89, 96–98]. A recent study in mice found that activation of the retina→vLGN→dorsal raphe (DRN) pathway is needed for the antinociceptive effects of green light [99]. It is worth noting that conventional photoreceptors, but not ipRGCs, are required for the antinociceptive effects of green light, suggesting that conventional RGCs can also mediate the antinociceptive effects of light (Fig. 3C). Interestingly, another study revealed that a circuit linking the visual cortex and the anterior cingulate cortex circuit plays a pivotal role in mediating the antinociceptive effects of green light (Fig. 3C) [100]. Given that the vLGN-related visual circuits are also needed for green light analgesia, it would be interesting to investigate the mechanisms underlying the interaction between vLGN-related visual circuits and the cerebral cortex.

### Circuit Mechanisms Underlying the Effects of Light on Sleep and Circadian Rhythms

Sleep is regulated by both homeostatic mechanisms and circadian rhythm [101]. Light can modulate sleep by entraining the circadian rhythm [2]. The SCN located in the hypothalamus is the central pacemaker of the circadian timing system [102]. Since ipRGCs project directly to the SCN, it was assumed that ipRGCs play a major role in mediating the effects of light on circadian rhythms. Consistent with this view, it has been found that specific ablation of ipRGCs results in the complete loss of circadian photoentrainment [103–106]. However, abolishing the intrinsic sensitivity to light of ipRGCs by removing the gene encoding the photosensitive protein melanopsin does not significantly affect circadian photoentrainment [107, 108]. Given that ipRGCs also receive light signals transmitted by rods and cones, it is plausible that rods/cones phototransduction *via* ipRGCs is sufficient for circadian photoentrainment. In support of this hypothesis, studies have shown that light does not regulate the circadian rhythm tested in the wheel-running test in mice lacking both rods/cones and melanopsin [109].

In addition to the SCN, other brain regions can also mediate the effects of light on sleep. It has been reported that sleep-related brain regions, such as the ventrolateral preoptic area (VLPO) and the superior colliculus (SC), are directly innervated by ipRGCs [66], and the changed neural activity in the VLPO and SC might mediate the acute light-induced sleep at night in mice [110, 111]. In addition, Zhang and colleagues found that acute dark exposure during the daytime induces wakefulness in mice by activating a retina→SC→VTA pathway [112]. They demonstrated that a dark pulse administered during the daytime disinhibits VTA dopaminergic neurons by inhibiting SC GABAergic neurons, which consequently leads to increased wakefulness and reduced sleep [112]. Thus, both circadian-related and circadian-unrelated mechanisms underlie the modulatory effects of light on sleep.

### Circuit Mechanisms Underlying the Effects of Light on Brain Development

The light sensation is crucial for brain development [113]. For example, raising animals in complete darkness or depriving one or both eyes at early developmental stages leads to significant shrinkage of thalamic axonal arbors in the visual cortex [114]. In 2014, Yu and colleagues found that dark rearing from birth reduces the excitatory synaptic transmission in multiple sensory cortices, and this impairment can be rescued by elevating the release of oxytocin from the paraventricular nucleus (PVN) through an enriched environment during the early days of dark rearing [115]. These findings suggest that the neuropeptide oxytocin is a key molecule in mediating the effects of light on brain development. However, the visual circuits that mediate the effects of light on oxytocin secretion still need to be determined. It is well established that ipRGCs become light sensitive much earlier than rods and cones, and play a pivotal role in mediating the earliest light sensation in mammals [116]. This suggests that visual circuits associated with ipRGCs might be important for mediating the effects of light on brain development. Consistent with this, a recent study by Hu and colleagues found the ipRGC→supraoptic nucleus (SON)→PVN pathway mediates light-promoted brain development [117]. They demonstrated that light-induced activation of ipRGCs increases the release of oxytocin from the SON and PVN into the cerebrospinal fluid [117]. Importantly, they found that a lack of ipRGC-mediated, light-promoted early cortical synaptogenesis compromises learning ability in adult mice [117]. These results provide new insight into the circuit mechanisms of the impacts of light on brain development.

In addition to the development of learning, early light sensation also influences non-photic circadian entrainment. Fernandez and colleagues established a time-restricted feeding model in mice by limiting food access to a 7-h period (ZT4-ZT11) [118]. They found that the innervation of ipRGCs at early postnatal stages influences IGL neurons that express neuropeptide Y, guiding the assembly of a functional IGL→SCN pathway [118]. Furthermore, they demonstrated that ablation of ipRGCs during early postnatal stages alters the connectivity between the IGL and SCN, reduces the expression of neuropeptide Y in the SCN, and results in decreased food-anticipatory activity in adult mice [118]. This result provides direct evidence that light sensation in the early postnatal period affects entrainment to time-restricted feeding through the ipRGC→IGL→SCN pathway.

### Circuit Mechanism Underlying the Effects of Light on Metabolism

Public health studies have shown that a disrupted circadian rhythm is highly relevant to metabolic diseases, including obesity [119, 120], diabetes [121], and cardiovascular disease [122]. Animal studies have also found that a circadian rhythm disrupted by varying light patterns affects metabolism [123, 124]. For example, long-term exposure to light at night alters internal hormonal rhythms [125–127] and thereby influences glucose metabolism [128, 129]. These studies suggest that light regulates metabolism by influencing circadian rhythm. In addition to regulating metabolism by affecting circadian rhythm, Meng and colleagues found that acute light exposure decreases glucose tolerance in mice through a retina→hypothalamus→brown adipose tissue (BAT) pathway [130]. They demonstrated that ipRGCs directly innervate vasopressin neurons in the SON, which project to the PVN and then to the GABAergic neurons in the solitary tract nucleus, and eventually to BAT. Light-induced activation of this pathway blocks the adaptive thermogenesis in BAT, thereby decreasing glucose tolerance. Moreover, they found that acute light exposure (400 lx) during the daytime or nighttime also decreases glucose tolerance in humans, and these effects are probably involved in the inhibition of BAT-mediated adaptive thermogenesis. These results unveil a visual circuit linking the retina and BAT that mediates the effect of light on glucose metabolism. It would be interesting to test whether a light therapy paradigm targeting this visual circuit can alleviate the symptoms of metabolic disorders, such as obesity.

In addition to regulating metabolism through specific visual-related circuits, Richard A. Lang’s group found that light also regulates the adipose tissue-mediated adaptive thermogenesis through two non-visual-related circuits. In one study, they found that blue light directly activates encephalopsin (OPN3, a blue-light-responsive opsin) located in mouse adipocytes, which in turn increases thermogenesis [131]. In another study, they found that violet light (380 nm) directly activates the deep brain photosensitive protein OPN5 in the hypothalamic preoptic area, and then suppresses BAT thermogenesis [132]. These opposing activities of OPN3 and OPN5 on thermogenesis raise the hypothesis that non-visual-related circuits decode light information to help the organism to maintain homeostasis through calibrating BAT activity appropriate to the time of day.

## Conclusions and Prospects

Growing evidence derived from both clinical trials and animal studies supports the beneficial effects of light therapy on multiple brain functions, including mood, cognition, sleep, and pain. However, the clinical application of light therapy has been faced with difficulties due to the unclear mechanism of action. As noted above, due to advances in research technologies, tremendous progress has been made in deciphering the circuit mechanisms of the effects of light on brain functions, such as mood, memory, pain perception, sleep, circadian rhythm, brain development, and metabolism. Several dedicated neural circuits have been demonstrated to mediate the beneficial and negative effects of light on brain functions. This experimental evidence not only provides a new theoretical basis for the application of light therapy in populations but also provides a new idea for developing novel light therapy strategies targeting specific visual circuits. Despite all these exciting advances, several key questions remain to be addressed.

Multiple key components of the neural circuit for non-visual light signal processing have been identified in the vLGN/IGL. However, an understanding of the vLGN/IGL-related circuits for light signal processing is not complete. For example, we found that vLGN/IGL neurons co-release GABA and glutamate and can be co-labeled by several traditional neural markers [91]. This suggests that vLGN/IGL neurons cannot be accurately classified by traditional neuron classification criteria. Fine-typing of vLGN/IGL neurons by single-cell transcriptome sequencing, thus revealing the neural connections and functions of different vLGN/IGL neural subtypes, is of great importance for further understanding the circuit mechanisms underlying the modulatory effects of light on brain functions.

Different light patterns can have opposite effects on the same brain function. For example, daytime bright light exposure may have antidepressant effects, while irregular light exposure may increase depression. It is important to establish new light therapy paradigms that can effectively activate specific visual circuits based on the unique light response properties of different visual circuits. This is crucial for the development of novel therapeutic strategies targeting the visual system to improve neurological diseases.

Finally, our knowledge of the effects of non-visual stimuli on the visual system is still limited and requires further investigation. For example, can changes in mood modulate the morphological and physiological properties of certain visual circuits? Can these effects impact the efficiency of light therapy? Systematic investigation of these questions will not only help to further understand the neural mechanisms of light therapy but also provide guidance for the development of new light therapy strategies.
